# Online art therapy pilot in the Western Isles of Scotland: a feasibility and acceptability study of a novel service in a rural community

**DOI:** 10.3389/fpsyt.2023.1193445

**Published:** 2023-05-22

**Authors:** Ania Zubala, Nicola Kennell, Catriona MacInnes, Marion MacInnes, Martin Malcolm

**Affiliations:** ^1^Institute of Health Research and Innovation, University of the Highlands and Islands, Inverness, United Kingdom; ^2^Research and Development, Green Tree Arts, Kinbrace, United Kingdom; ^3^Research and Innovation, NHS Western Isles, Stornoway, United Kingdom

**Keywords:** art therapy, online psychotherapy, mental health, online intervention, pilot feasibility study, rural community, audio image recordings, service evaluation

## Abstract

**Introduction:**

Art therapy, despite being an evidence-based, safe and acceptable intervention, is not widely available to clients in Scotland. Online delivery has the potential to expand the reach and accessibility, but special considerations need to be given to designing successful online art therapy services, due to the unique emphasis on the role of an image and artmaking alongside the therapeutic relationship.

**Methods:**

A pilot online art therapy service was developed and delivered in the Western Isles of Scotland to individual adult clients wishing to increase their psychological wellbeing. This research aimed to assess feasibility and acceptability of the novel service, identify enablers and challenges in setting up and delivering the service, explore participants’ expectations and experiences of art therapy and identify any impacts of the service. Mixed-method evaluation incorporated questionnaires, focus groups, interviews and Audio Image Recordings (AIRs). Findings were grouped into themes across several key areas: service setup, research procedures, intervention design and impacts and insights. Recommendations were developed for the first three areas and the last section presents indications of change and gives voice to client experience primarily.

**Results:**

Online art therapy was described by clients as a judgement-free zone which allowed freedom to experiment, express, feel and immerse themselves in the creative flow. Other benefits included readiness to accept emotions, increased understanding of self and others and being able to see things from a new perspective. Clients recognised the unique nature of art therapy in relation to other psychological treatments and valued the freedom of expression it offered, including the non-verbal.

**Discussion:**

This project demonstrated that online art therapy is not only a feasible an acceptable intervention, but potentially also a powerfully impactful one, capable of instilling a positive change in a surprisingly short period of time. Exploring ways to expand current and introduce new art therapy services is highly recommended. Refinement of an intervention design, tools and research procedures is recommended through further feasibility studies of a larger scale.

## 1. Introduction

Art therapy is “a form of psychotherapy that uses visual and tactile media as a means of self expression and communication” (1). Creative self-expression and therapeutic relationship are the essential therapy tools used to address a range of emotional and psychological challenges that might be difficult or distressing. Due to not relying entirely on verbal communication, art therapy is a particularly suitable form of psychotherapy for those clients who find verbal expression difficult or who may be looking to expand their ways of communicating thoughts and emotions which are hard to verbalise. Since art therapy has both curative and preventative potential (2–4), clients who do not have specific psychological difficulties might use art therapy to increase their quality of life and enhance wellbeing. Therapeutic approaches integrating elements of different psychological theories are often considered most suitable in addressing individual clients’ needs (5, 6), while flexibility and person-centredness are at the core of most art therapists’ practice.

Art therapy is an evidence-based intervention with well-documented positive impacts on a wide range of client groups with diverse psychological difficulties. Systematic reviews have established multiple benefits of art therapy for common mental health conditions, including depression and severe anxiety (7–13), and demonstrated its success in addressing psychological impacts of long-term or life limiting physical health conditions (14–16). Evidence is also growing of sustained benefits of art therapy (17, 18) and of it being a highly acceptable form of treatment (19–21).

There are encouraging indications from Australia (4), Canada (22), and the US (24, 25) that art therapy might offer a valuable and relevant support for clients of all ages living in rural areas. However, despite growing evidence of its benefits and acceptability, art therapy is rarely available in more remote locations in Scotland, with existing art therapy services predominantly centred around urban areas. Limited number of practitioners, geographical challenges and time and financial burdens of travel mean that individuals in rural areas experiencing psychological distress, including patients with life-limiting medical conditions, miss out on this valuable intervention (26).

Art therapy discipline has increasingly welcomed opportunities offered by digital technology for both online therapy and digital art making (26). Most recently, similarly to other mental health practitioners in Scotland (27, 28) and beyond (29–31), art therapists embraced digital technologies enabling them to connect remotely with clients during the Covid-19 pandemic and ensuring continuity of treatment (32). A survey of UK-based art therapists gathered practitioners’ early experiences of their transition to online practice, with as many as 90% art therapists reporting that they intended to honour their clients’ preferences for mode of delivery and would expect to be working online at least to some extent in the future (33).

Growth in remote care in mental health provision seems inevitable (34). Potential benefits for clients living in rural and more remote areas reach beyond overcoming geographical distances and increasing access to services. There are indications that some clients might engage more willingly with therapy provided at distance and might feel more empowered and in control of own recovery (26, 35, 36). Online delivery has also additional benefits of increased privacy, which might be particularly welcomed by clients in small communities.

Online psychotherapies can be as effective as those delivered face-to-face (37) and therapeutic process can develop to at least the same extent online as in face-to-face therapy (38, 39). However, offering online art therapy safely and efficiently requires certain adaptations to practice and, at the same time, presents unique opportunities for the therapeutic process (25, 26). In case of art therapy, there are indications of both limitations and affordances of it being delivered online, some shared with other psychological therapies and others specific to the nature of art therapy practice, in particular work with art materials, space for storing artwork and opportunity to share the creative process (33). Despite growing public interest in online health interventions and rapidly progressing expansion of art therapy practice into online realm, research into therapeutic mechanisms and client experiences of therapy is still limited and much needed to inform practice.

Art therapy services for adults in the Western Isles of Scotland have been offered to a modest extent via the Western Isles Council, Taigh Chearsabhagh (Museum and Arts Centre), and privately. These services have been welcomed in the community but are limited in their reach and accessibility. The current pilot service was developed in response to this need and commissioned by the NHS Western Isles (NHSWI), aligning with its European INTERREG mPower social prescribing project aims, including investigating the potential for online wellbeing-focused interventions in the remote rural community. Feasibility and acceptability of the service were the key concerns in its evaluation, with focus in particular on online mode of delivery in the rural context of the Western Isles.

In line with intention-to-treat principle, the project’s primary aim was to deliver online art therapy to adults who might need it, regardless of if they wished to take part in evaluation. The sample was meant to be small and data collection deep rather than wide in order to identify any supportive and challenging mechanisms in the process of setting up, delivering and evaluating of an online art therapy service. Lessons were expected to be relevant to developing other online health and care services and psychological treatments in particular. Establishing acceptability and feasibility of research-related processes was intended to guide future study designs in art therapy and related disciplines.

This research aimed:

- to assess feasibility and acceptability of the novel online art therapy service,
- to identify enablers and challenges in setting up and delivering the service,
- to explore participants’ expectations and experiences of art therapy and
- to identify any impacts of the service (on the participating individuals and the wider community).

This paper outlines the process of establishing a pilot online art therapy service within an island community, presents key findings from a feasibility study, focusing primarily on client experience and proposes recommendations for future practice and research.

## 2. Materials and methods

**Figure 1:**
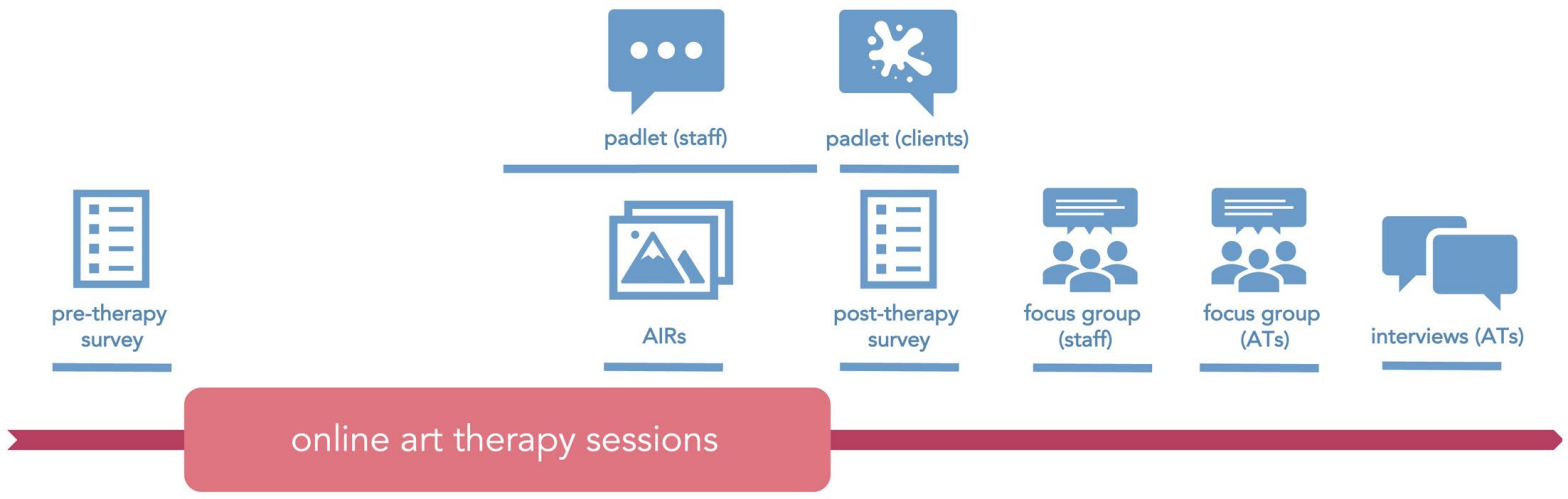
Research methods and their timeline in relation to the intervention.

Evaluation of this project employed mixed methodologies within qualitative and quantitative paradigms, as well as arts-based elements. Due to key interest of this project in participants’ experiences and its pilot nature, more weight was given to qualitative methods. A variety of methods was used to allow for triangulation of findings across different groups of participants and within them (40). A mix of methods was considered most suitable for capturing change and the complex and unique nature of art therapy practice. Stakeholders invited to share their experiences of the service included: (a) staff assisting in setting up the service and referring clients to therapy, (b) art therapists, and (c) art therapy clients (recipients of the intervention). At least two different methods of capturing experiences were used within each group (Figure 1).

Ethical approval for the work was granted by the University of the Highlands and Islands on the 15th May 2021 (ref. ETH2021-1176).

### 2.1. Intervention design

The online art therapy pilot service utilised some of the procedures and infrastructure already in place in the Western Isles, primarily through the NHS Western Isles European INTERREG mPower project (41), including alignment with mPower referral processes, where appropriate, and involvement of community and digital navigators in referrals and ongoing support. Information about the project and referral criteria were distributed among third sector groups in the Western Isles, who were also invited to a dedicated online session, introducing the project and offering opportunity to raise concerns and shape the referral process. Eight community-based organisations were consulted and four expressed interest in the project, of which one reported no interest from clients and three went on to referring clients to the service.

Therapy was offered by two fully qualified and experienced art therapists, who had practised extensively with a range of client groups and in a variety of settings and were particularly familiar and experienced in working with rural communities and clients who lived more remotely. The therapists were closely involved in setting up the service, were consulted on study design to limit potential interference with the therapeutic process and facilitated specific research-related elements as part of their role. They met regularly for peer and clinical supervision specifically for the purpose of this project.

Art therapy was offered to clients on an individual basis, as a block of eight 1-h weekly online sessions. An additional ninth session was offered to each client for the purpose of making an Audio Image Recording (see *Participants and methods: Clients*). This specific session was scheduled between the seventh and the eight (last) therapy session, to allow time for processing and consolidating of any new material arising from the experience. Art therapists were free to use any art psychotherapeutic approach according to individual clients’ needs and their professional expertise and judgement.

Therapists and clients connected via Attend Anywhere, a video consultations platform approved for use within the NHS and utilised by Near Me services in Scotland (42). Clients could use their own digital devices to connect for their therapy if they wished. They were also provided with iPads complete with Apple Pencils and a pre-loaded Procreate application on each device for the duration of their therapy, which were setup and provided by the NHSWI IT department. These could be used to connect with the therapist, share images and/or for digital art making. Sets of traditional (physical) art materials were also provided to clients free of charge.

### 2.2. Participants and methods: clients

The service was intended to be inclusive and suitable for adults of all ages, experiencing a range of emotional difficulties, regardless of formal mental or physical health diagnosis. Detailed recommendations for referral were developed by the art therapists and the researcher and shared with referring organisations. Essential criteria included, for example, willingness to work creatively in a supportive (virtual) environment, to reflect on experiences and feelings and to try using arts media to communicate and express emotions. While mental health diagnosis was neither essential nor excluding, it was recognised that online art therapy in this pilot might not be suitable for some clients with complex mental health diagnoses (e.g. psychotic illness, personality disorder, current PTSD symptoms) or current suicidal intent. Referrals could not be accepted for clients who engaged in psychotherapy at that time. Art therapists were not only guided by the referral criteria but also worked closely with the referrers, with each other and with prospective clients to establish if art therapy might be a suitable option for each individual.

Adults who were offered a place in the online art therapy service were at the same time invited to take part in the research. Participation was entirely voluntary and the clients’ decision to participate (or not participate) had no consequences on their involvement in therapy. Participants were free to withdraw from either part of the project, including therapy sessions, at any point in time. Research methods were carefully chosen and adapted for the use in this project, resulting in robust but relatively non-intrusive data collection procedures.

Clients who chose to take part in the research were asked to complete online questionnaires (hosted on the JISC Online Surveys platform) at two points in time: prior to starting online art therapy and on its completion. Both online surveys included two short self-reported psychometric measures (Warwick-Edinburgh Mental Wellbeing Scale and WHO-5) and a questionnaire devised for the purpose of this project, asking about participants’ expectations (pre-therapy) and the actual experience of therapy (post-therapy). In order to ensure anonymity, participants were asked to choose a memorable nick name that would allow for linking responses from both surveys. On completion of their course of therapy, participants were also invited to share their experience through artwork, which they could upload to a dedicated online Padlet space.

Clients who wished to create their Audio Image Recordings (AIRs) with their art therapists, did so in their second last therapy session and consented to their videos/images being shared for research and educational purposes. AIR is a unique art therapy-specific evaluation method, involving a client sharing an image (or a couple of images) of their artwork and responding to questions asked by their art therapist, which is captured in a simple voice-over-image video (43). This arts-based method serves multiple purposes: (a) it supports clients and therapists to have a somewhat structured reflective conversation on the therapy process, (b) it allows clients to hear themselves describing their experience, which can be therapeutic in itself, (c) with client permission it may be used for research purposes and to raise awareness of art therapy among the wider public. Recordings captured by the art therapists were subsequently edited by the researcher and uploaded to a private YouTube channel, accessible through shareable weblinks. Clients were offered an option to have their voices electronically modified and care was taken to remove any potentially identifiable information.

### 2.3. Participants and methods: staff

Staff involved in setting up and delivery of the pilot service, including NHSWI mPower staff and Third Sector group partners, were invited to take part in several stakeholder meetings prior to the start of the project. They also had an opportunity to communicate any observations and feedback regarding the project throughout its duration, via e-mail and a dedicated online Padlet space.

Staff were also invited to take part in an online focus group towards the end of the project, after the therapy had finished for all clients. The group discussion lasted 1.5 h, was facilitated by the researcher via MS Teams and recorded for the purpose of analysis. The focus group was conducted around the client journey through therapy—from referral to post-therapy situation. Participants were invited to share their honest feedback on various aspects of the service and particularly on what worked well, what did not and what they felt might be improved in similar projects in the future.

### 2.4. Participants and methods: art therapists

Having finished their art therapy sessions, both art therapists took part in a focus group-like discussion with the researcher followed by individual in-depth interviews providing a space to further explore the key themes arising from the group discussion. In addition to topics similar to those explored in the staff focus group, art therapists were invited to also reflect on issues specific to art therapy practice, including: (a) development of therapy process and therapeutic relationship in an online setting, (b) adaptations to practice required in online delivery, (c) insights into artmaking using digital media as part of therapy, and (d) suitability and therapy-related impacts of research methods, including in particular AIRs.

### 2.5. Data analysis: cross-group and intra-group themes

The questionnaires, focus group theme guides and interview schedules were designed to gather information and capture participants’ experiences on a number of pre-defined topics, which were considered pivotal for the process of service development. All methods also provided dedicated ‘open spaces’ for any off-topic comments to be shared (e.g. free text items in the questionnaire, Padlet available throughout the project, invitation to share further insights following interviews and focus groups), allowing unintended effects or surprising insights to be captured.

Thematic analysis of data collected through this flexible design resulted in a set of themes shared across the different groups of participants and themes specific to a particular group experience, as well as, essentially, individual opinions and experiences. The findings pertaining to cross-group experiences are presented first, followed by findings pertinent to experiences of specific participant groups. Direct quotes are presented in *italics* and accompanied by an indication of a participant group source: C for clients, S for staff (including referrers), AT for art therapists. Any potentially identifiable information was either removed or concealed (e.g. gender indicative pronouns replaced).

## 3. Results

Eight individuals were accepted for online art therapy offered via this pilot service, all of whom also took part in some or all the research procedures they were invited to. Of the six clients who completed the pre-therapy survey, four went on to also complete the post-therapy survey. Two other clients who chose not to complete the pre-therapy survey decided to do so post-therapy. Six clients agreed to do their AIRs with the therapist. However, recording did not go ahead for one client due to issues with technology and timing, while one client expressed a wish to do a second recording in response to their initial AIR, resulting in six AIRs being produced, of a length between about 7 and 12 min each. Five images were contributed to the Padlet online area.

Four members of staff involved in setting up and delivery of the pilot service took part in an online focus group and one staff member who was not available at that time agreed to take part in an interview instead. Both art therapists took part in a focus group and in individual interviews.

In total, 15 participants took part in the research: five members of staff, two art therapists and eight clients.

The eight clients were all females, of different ages (two were under 30, four between 31 and 60, and two over 61 years old). Although not necessarily confirmed by formal diagnoses, their predominant mental health presentations, as assessed by the art therapists, included: low mood (all clients—including at least three individuals with depression), anxiety (five clients), adverse childhood experiences (six clients, with likely linked trauma for some) and chronic psychosomatic pain (three clients).

### 3.1. Service setup

#### 3.1.1. Preparation

Staff perceptions on the amount of time spent on preparation to the project differed with some people feeling that not enough time was allocated to the early stages and others feeling that they had spent long time waiting for things to happen before they could move on in their roles and tasks. In effect, activities leading towards the start of the project were not always adequately co-ordinated in time, requiring human effort and flexibility to mitigate potential negative consequences (e.g. sessions with clients arranged before equipment was delivered).

Due to the restricted timeline of the project, all clients started their therapy around the same time, which the art therapists felt was *a lot to suddenly do, a lot to hold emotionally (AT)*. They, however, recognised that the workload felt more feasible in the online therapy setting than it would have been in a face-to-face situation as it allowed for more flexibility in arranging sessions and removed the need to travel between spaces. Art therapists felt that the clients would have also benefitted from longer time to ‘settle in’ therapy. In this pilot, the referrers and digital navigators supported clients in the early stages (prior to therapy) with practical arrangements around technology, with art therapists having e-mail contact only. Art therapists felt, however, that they would have liked to meet their clients prior to starting therapy sessions and dedicate more time for laying foundations for working together so that time in the early sessions can be used more effectively:

That holding is really important, and because you're not on the ground, you're not doing that, someone else is bringing materials, the iPad, (…) an art therapist doesn't have control over that aspect in this case. And so, we're coming in remotely, at that point, and then trying to make a beginning, which takes time. (AT)

Despite the above challenges, the service was set up in time for the intended course of therapy to take place and concerns about time and communication in the preparatory stages of the pilot *settled once the activity [art therapy] began (S)*, which was described by one person as *lots of surprises in the set-up but not the delivery (S)*. One member of staff reflected: *Practical stuff about the set up was difficult, but the benefit outweighs all that (S)*.

**Recommendations: Preparation**

Allocating sufficient time for setting up an online art therapy service is crucial, particularly in its early stages. Realistic timelines, roles and responsibilities clearly defined and clear guidance on tasks and procedures are important for ensuring success from the start. Extra time should be allocated to responding to any unforeseen adaptations, if required. Setting up regular opportunities to meet for all stakeholders is recommended to aid communication, to resolve issues early on and provide clarity on roles as well as awareness of individual needs and the type of support staff may require. Weighting tasks according to priority and considering chronological order in which activities need to happen would help identify those that need to be attended to early in the process (e.g. arranging tender for therapists if not already in place).

#### 3.1.2. Referral process

Some third sector groups seemed to be cautious about referring service users to the pilot, perhaps not having enough *understanding of what art therapy really was about (S)*, despite the information and flyers provided. There were indications that some referrers were seeing art therapy as similar to art activities and concluded that their clients who had been already involved in art classes might not have further need. One potential referrer had concerns about burdening their clients with *something additional, something new, irrespective of how beneficial that might be (S)*. An art therapist felt that it was common for other professionals not to be familiar with art therapy and discussion was helpful: *Part of the work is just discussing it. People over time get an idea of what we do and do not do or what we can best achieve (AT)*. One referrer, however, shared that they had been *looking for art therapy for [their] clients for years (S)*.

There was also some confusion about the referral criteria with staff reporting uncertainty about the reasons why some referrals were not considered suitable by the art therapists, despite the inclusion criteria provided and having met with art therapists to discuss. In particular, there did not seem to be a shared understanding as to why certain individuals with dementia were not offered therapy, which in that case was primarily due to the time-limited service which the art therapists felt might not allow space and time to accommodate the unique needs of people living with dementia. Referrers also acknowledged their limited confidence in assessing client suitability for art therapy, e.g. *I was surprised about one referral as I thought that would not be [their] thing at all and [they] probably got more out of it than anybody (S)*.

Participants generally felt that it was important that *the referral form was coming through somebody they [the clients] knew and were already involved with (S)*. Referrers felt responsible to some extent for their client’s experience, at times supported them to settle into their therapy and in some cases throughout the process: *I wanted to make sure that they [clients] succeeded, that they had everything they needed and were comfortable (S)*. For the art therapists, the support from the referrers was important in creating a safe environment for clients in the time-limited therapy.

**Recommendations: Referral process**

Involving NHS-based partners and engaging an independent clinician (e.g. clinical psychologist) in the referral process could increase referring organisations’ confidence in getting involved. A risk assessment procedure shared across partners in the project may be helpful in addition to the art therapists’ own. It is important to arrange a discussion within the team about clinical governance in private practice and allocate sufficient time for stakeholders to meet with art therapists and raise any concerns they may have. Reconsidering ways of sharing information about art therapy practice may be needed to ensure a shared understanding. It is important to agree a good triage system to avoid any confusion around referral criteria and have procedures in place for communicating with individuals referred.

#### 3.1.3. Technology

Delivery of the online art therapy via the Near Me service required initial co-ordination of several parties, including NHS IT team, mPower staff and the therapists. Once the system was in place, however, only some minor technical issues were reported and supported by the digital navigators and, at times, by the referrers, with one referrer reporting that they had given their clients *a little bit of teaching beforehand (S)*. In one case, the referrer offered to assist the client in setting up the connection and accompanied them in person for the very beginning of the first session [*It is important that this support is in place to minimise anxiety and keep people engaged in the process (S)*]. The client and the therapist made special arrangements for subsequent sessions, *where [the client] would log in at an earlier time to check all the technology (AT)*.

Both therapists had a very positive experience of the platform in this pilot and reported that it offered smooth connectivity and functionality. It also seemed to have worked well for the clients (with some exceptions, generally resolved quickly with the support of digital navigators). However, even though the platform provided good enough quality connection between the clients and the therapists, its functionality is limited for use in art therapy. Art therapy practice requires artwork to be shared and for the therapist to observe the process of artmaking alongside client’s facial/body expression. To compromise for the lack of such inbuilt functionality within the platform, photographs of artwork were often shared via email and/or artwork was lifted to the camera for therapists/clients to see. One client remarked that *there was no way to show both [their] face and the art (C)*.

Arranging for the digital equipment (iPads) to be provided for clients was, again, time consuming and there were indications that some clients might have preferred to use their own devices, being more used to, for example, an Android operational system. Indeed, most clients reverted to using their own devices towards the end of their therapy. One art therapist felt that clients would appreciate some dedicated training in the use of the Procreate app: *It would be better to have someone that would actually go through Procreate with them (AT)*.

**Recommendations: Technology**

An online art therapy service demands reliable digital technology, including high quality Internet connection, to be in place for everyone well in advance of the actual therapy sessions starting. NHS emails need to be arranged early for art therapists, enabling contact with clients prior to the sessions. Sufficient time needs to be allocated for preparation, co-ordination and equipment setup and, essentially, for clients and art therapists to familiarise themselves with (likely) new to them systems. Training sessions should be available for therapists and clients who may need them – in using the platform and also in using app(s) and equipment for digital artmaking, should they wish to try this. Support arrangements need to acknowledge individual needs (e.g. wish to use own device) and varying confidence and familiarity with digital technology. A technology-focused check-in early in the therapy (separate from therapy sessions) can be arranged to identify any issues that may still need to be resolved. Finally, development of a bespoke digital platform better suited to art therapy practice may be considered.

#### 3.1.4. Research design

Aiming not to interfere with the therapy process, the research was designed in a way that protected clients’ anonymity as far as possible. That meant that the researcher did not have direct contact with clients and therefore the research element of the pilot relied heavily on staff and therapists. One staff member felt that clients would find it acceptable to talk with the researcher directly (either face-to-face or online), suggesting interviews as a good option for those clients who might be worried about digital records being kept safely and those struggling with questionnaires.

Six clients who completed the post-therapy survey found the questions easy to answer [*it was not a big deal. (C)*] and *perfectly fine (S)*, with one participant also saying that they were useful as a reflective tool [*made me happy to reflect (C)*]. Two clients who completed the pre-therapy survey decided not to take part post-therapy. Even though four clients completed both surveys, responses could be linked for three clients only due to one participant failing to use the same nickname in both surveys. One referrer indicated that some clients found it difficult to answer the self-report wellbeing questions (WEMBS and WHO-5 scales), which presumably might have discouraged them from doing it again post-therapy:

One client with [MH condition] could not face completing psychometric scale, felt it was overwhelming, could not decide how [they] felt. (…) One client with [MH condition] found it a bit daunting, [they] obsessed about [their] answers: do I feel great? or do I feel a little bit great?… Because people do try to answer honestly… (S)

Six clients agreed to do their AIRs and one did not, explaining that they felt confused and hesitant about doing this and *did not feel comfortable showing [their] art (C)*. One recording did not go ahead due to technical difficulty. Three clients described a positive experience of recording their AIRs with one person saying it was *brilliant (C)* and another feeling that through doing this they were *able to explain how [they] found the whole experience [of therapy] (C)*, and one participant reflecting: *It was a safe and understanding experience and [the art therapist] guided me through it very well and thanked me for my input (C)*.

Referrers also observed the positive impact that recording their AIRs had on clients, with one person noting that the recording *made [the client] realise there was something going on and [they] needed to get to the bottom of it (S)*. One person also observed that some clients seemed to have been *quite proud of the recording that they did, it was a real boost of confidence (S)*. One staff member recognised the value of AIRs as a research method: *When people see the videos [AIRs], that is where the real feedback is, I think (S)*. Art therapists felt that doing AIRs was an important experience for their clients and they themselves found them to be a valuable reflective tool, enhancing their practice [*For me it was quite a turning point in thinking about future work really in terms of capturing things and actually being able to use that recording as a reflective tool (AT)*]. One art therapist said they were *struck with how generous everybody was, just wanting to do this knowing it will help someone else to maybe start thinking about doing art therapy (AT)*. Clients valued the opportunity to review their recordings with the therapists in their last session together, which in one case resulted in the client recording their second AIR, in response to the impression that seeing their first recording has made on them. Both the art therapist and the client found the experience very rewarding:

It was such a powerful thing for [the client] and such a powerful reaction to [their] artmaking and what [they] saw in [themselves] that to make that second one felt so important, because to me that really captured that whole powerfulness of the therapy, of what [they] were doing. (AT)

As I was listening, it was a really good insight. I took a step back and I could see a distorted sight of me. It was a good experience to have to think about actually what I have deep inside me that I could bring out (C).

**Recommendations: Research design**

It is recommended that future research continues to utilise mixed methods for capturing the complex multilayer nature of art therapy practice. Study designs should be guided by the intention-to-treat principle, which in larger studies should not compromise recruitment if, for example, the control group is offered therapy at a later stage. Audio Image Recordings should be seen as a valid method in art therapy research, capable of capturing insights of a depth difficult if not impossible to achieve via other methods. Sufficient time and resources need to be allocated for editing and sharing of AIRs with the wider team. While questionnaires are helpful and generally acceptable tools, the use of psychometric scales may need to be reconsidered with some client groups and/or optionality in answering questions offered. Pre-testing of mental wellbeing measures is recommended in order to identify scales that are likely to work best in specific contexts. A reliable system for linking responses should also be in place. Anonymity might not be in fact as important to art therapy research participants as an opportunity to speak with the researcher directly and in person interviews should be considered, offering an additional space for reflection.

#### 3.1.5. Intervention design

##### 3.1.5.1. Traditional and digital artmaking materials/tools

There was a shared feeling among staff that the clients appreciated the opportunity to use the variety of (often new to them) art media, which were not necessarily readily available to buy around where they lived other than online [*Experiencing all the different [art] materials that I have not used before has been great (C)*]. Art therapists felt that for some clients there had been *a real excitement about the material packs coming (AT)*, which *set [them] off on a positive foot (AT)*. Art therapists also felt that for some clients having the art materials provided was an important part of the therapy process and particularly helpful in the early stages of therapy:

[Some clients] felt something quite nurturing about this pack that had been given to them and it felt quite important, and I noticed there was a real sense of looking after the materials and real pride in their packs as well. (AT)

One staff participant felt that digital artmaking *was either very loved or very hated (S)* by clients. Of the six clients who completed the questionnaire following their art therapy, three tried making digital images in their art therapy and three did not. One of those who tried reported that they did not enjoy it as they *liked to be hands on and have the materials in [their] hand to touch (C)*. Two clients particularly enjoyed making digital art, particularly the variety of (digital) arts media available through the app and ease of use: *I really enjoyed the variety of tools I was able to experiment with, it was really good fun and easy to share (C)*.

One of the art therapists noticed benefits of digital artmaking for one client in particular, *who felt less confident with the art materials (AT)*, observing that it aided their readiness to be creative and experiment: *The iPad actually was easier and it was convenient. It was practical, and it tapped into something personally for [them], I think, as a tool (AT)*. That client was hoping to make arrangements to be able to continue using the Procreate app on own device beyond the duration of therapy.

Both art therapists enjoyed using iPads for digital artmaking in sessions with clients and felt that, although it offers a fundamentally different experience, it is an appropriate arts medium for use in art therapy, certainly for some clients. One of the art therapists found themselves engaging in digital artmaking for the purpose of ongoing reflection on their clients’ therapy process, valuing it as a useful tool in professional practice, particularly for keeping and revisiting records of client progress:

I made an image after every single session that I did and that was brilliant. I loved doing that. I felt it was so easy drawing on the iPad. I found that really freeing as well as being very containing. And once you turn it off, it's gone almost, but it was there, so for me there was a really good record. (…) I would be looking at them all at the sequence and it just really helped to work. (AT)

##### 3.1.5.2. Connecting online

Art therapists appreciated that online working allowed for more flexibility in arranging times to meet with their clients and adapting more easily to their needs, which they felt the clients were valuing. This potential for adaptation extended to being able to offer options for clients to switch their camera off, if needed, and for them to have more control over sharing their artwork [*being able to offer that greater flexibility when needed is something I’m really valuing with the online working (AT)*]. Clients and participating staff generally agreed that the online mode of delivery was convenient and meant, for example, reduced costs of travel and not having to arrange child care [*invaluable, because there was no travel time required, not as much arrangements had to be made (S)*].

Staff also felt that online delivery was important for removing certain barriers to engaging, particularly for those clients who *might not be entirely comfortable being out (S)* or those who had lost *a lot of confidence over the last few years (S)* due to the Covid-19 pandemic [*it is important they are given the choice to do things online (S)*]. Three clients indeed referred to the impacts of the pandemic, noting that online delivery is a safer option and that having the choice to connect online might help those who struggle with interpersonal interactions, either as an effect of the pandemic or in general. One client imagined that connecting online might make them feel relaxed, while another appreciated that it would give them more time to prepare.

In their responses to the questionnaire, four clients indeed confirmed that online delivery was an important factor in making the decision to take part in art therapy, for three participants that was not important and one participant was not sure. One client shared with their art therapist that they *would not have come face to face* (…) *this opportunity to engage online was what made [them] do it. (AT)* While it seems that for the majority of clients connecting with the therapist online, at least to some extent, was a preferable option, one client shared that they found it difficult *not having the therapist in the same room (C)* and that they would have preferred *to be away from the home setting and any issues you have going on (C)*.

##### 3.1.5.3. Length and structure of therapy

The structure of the therapy and its pre-defined duration in this pilot presented both advantages and challenges for all involved. While art therapists found the clarity of the structure to be helpful in some ways, they agreed that having an option to extend therapy would have been helpful for most of the clients who took part:

If there had been another block of time (…) no doubt that each person would have continued to use the space and wanted to use the space. And in terms of the development of what they were doing, I think that would have unravelled further. (AT)

A similar view was shared by a member of staff who felt that *if the therapy could have gone on longer that would be such a benefit to [the client] (S)*, as [they] were *really getting into it when it stopped (S)*. One client in particular also felt that *the allotted number of weeks wasn’t enough (C)*, particularly as they found it difficult to commit to the set times during the week. The same client also *did not feel comfortable with the therapist only being available during the session and not at all outside of it (C)*, highlighting that the fundamental frame of art therapy practice might not be suitable for all clients.

**Recommendations: Intervention design**

Offering art therapy clients a choice of art materials is recommended, including digital artmaking tools for those willing to try or not comfortable with traditional art media. Ideally, length of art therapy would be agreed with clients on individual basis and with regular reviews. Time-limited art therapy, however, can still be beneficial, particularly in an online setting where the therapeutic process seems to develop more intensely for some clients. Art therapists should consider aspects of practice specific to online settings not only mirroring face to face arrangements in virtual spaces but also reflecting on unique opportunities that online work opens.

### 3.2. Development of the therapeutic process in an online and time-limited therapy

Connecting online from private spaces seemed to have implications for the development of the therapeutic relationship. One art therapist reflected: *There is an intimacy about seeing people in their own homes and I think it becomes a shared responsibility really to set up the frame (AT)*. The importance of a comfortable, quiet and private space to connect from was not obvious for all clients at the beginning of their therapy [*not knowing what therapy is until they start, so not knowing what they need really in the beginning in terms of the space (AT)*].

The art therapists found that some of the practical challenges specific to online practice, like issues with connectivity and digital technology, could have been used with some clients, quite unexpectedly, to aid the therapeutic process. With one particular client, the therapist felt that the technological challenges prompted the client to find solutions and *actually showed a real resourcefulness in the person (AT)*:

[The connectivity issue] was quite a parallel in terms of the person themselves and how they manage things not working out. So actually we were able to utilize that theme of how they experience that, connecting up as part of our narrative in the session as well as in other situations, which was helpful. (AT)

One of the art therapists reflected how working in an online space, quite paradoxically, seemed to have intensified the experience of connection and strengthened the focus of their therapeutic work:

There's something about the kind of tightness and smallness of the space that sort of intensifies everything somehow, and it doesn't feel like a flat screen. When the connection is good, it feels very three dimensional, like a capsule. (AT)

Despite recognising that a longer time for therapy would have been beneficial for most clients, the art therapists did not find the time limit to be too restrictive for the therapeutic process to unfold. On the contrary, they felt that the frame might have at times supported progress and engagement in the process. Both therapists agreed that although *at the end it was starting to feel like a very short piece of work,* (…) *it was still possible to do a lot in a very short space of time (AT)* and they recognised *how much and how deep [the clients] all worked. (AT)*, despite the seeming brevity of therapy. Awareness of the limited time, and hence, the need to focus, might have in fact helped clients to make progress:

There was quite an awareness of keeping themselves [clients] safe, knowing that they had a limited time, but equally really wanting to maximize the opportunity that they had and absorb themselves in as much of that as they could (…) I don't feel it hindered the processes at all, but I think it was certainly a theme that was there. (AT)

### 3.3. Impacts and insights

#### 3.3.1. Expectations and the actual experience of online art therapy

Seven participants have not experienced art therapy prior to this pilot and were not offered it before and one participant who has taken part in art therapy previously, shared their positive experience:

My previous experience was extremely fulfilling, I was able to concretely work through a number of issues that had been really troubling me, it allowed me to improve upon relationships, take a step back and accept the feelings I’d been having in a safe environment. I felt validated and understood by my art therapist, her feedback and guidance was crucial to the therapy process. (C)

Three participants were not sure what to expect from the art therapy while others anticipated to be having a conversation with an art therapist and making art either within the sessions or in between sessions [*Talking about how I feel and showing how I feel through my art. (C); a good rapport with therapist (C)*; *An interactive, welcoming and supportive collaboration between therapist and patient. A safe space to explore various issues and learn to understand oneself and how we might better interact with the world around us as a result (C)*]. One participant expected to be offered topics to explore in their art and that the work would then be reviewed with the therapist. Participants highlighted that they appreciated the opportunity to try art therapy and two people expressed slight nervousness about starting, including related to lack of confidence in using digital technology. One person hoped that art therapy would equip them with tools to use beyond its duration: *I wanted to know how it worked so that I could give myself the tools to do it myself (C)*.

On committing to art therapy, the participants were hoping to achieve a range of benefits for themselves, including:

- opportunity to express, confront and accept emotions: *to talk through all my feelings and why I have them (C), to express and figure out why I was feeling emotions that I knew were affecting my everyday life (C), acceptance of uncomfortable feelings (C).*
- a way to release tension: *A form of relaxation and possibly some soul searching (C), to be able to relax and concentrate on it and feel happy (C), calm tools to relax and not panic about new things (C).*
- personal growth: *A safe space to explore and move on from things that hold us back in life (C), a better understanding of self (C), increased confidence (C).*
- a protected time for oneself and purpose: *time for me, give me a focus to do something (C).*

Of the six participants who completed the questionnaire following their art therapy, three felt that it was as they imagined, two were not sure and one person said that it was not what they expected purely because *not having done it before [they] did not really know what to expect (C).*

**Figure 2:**
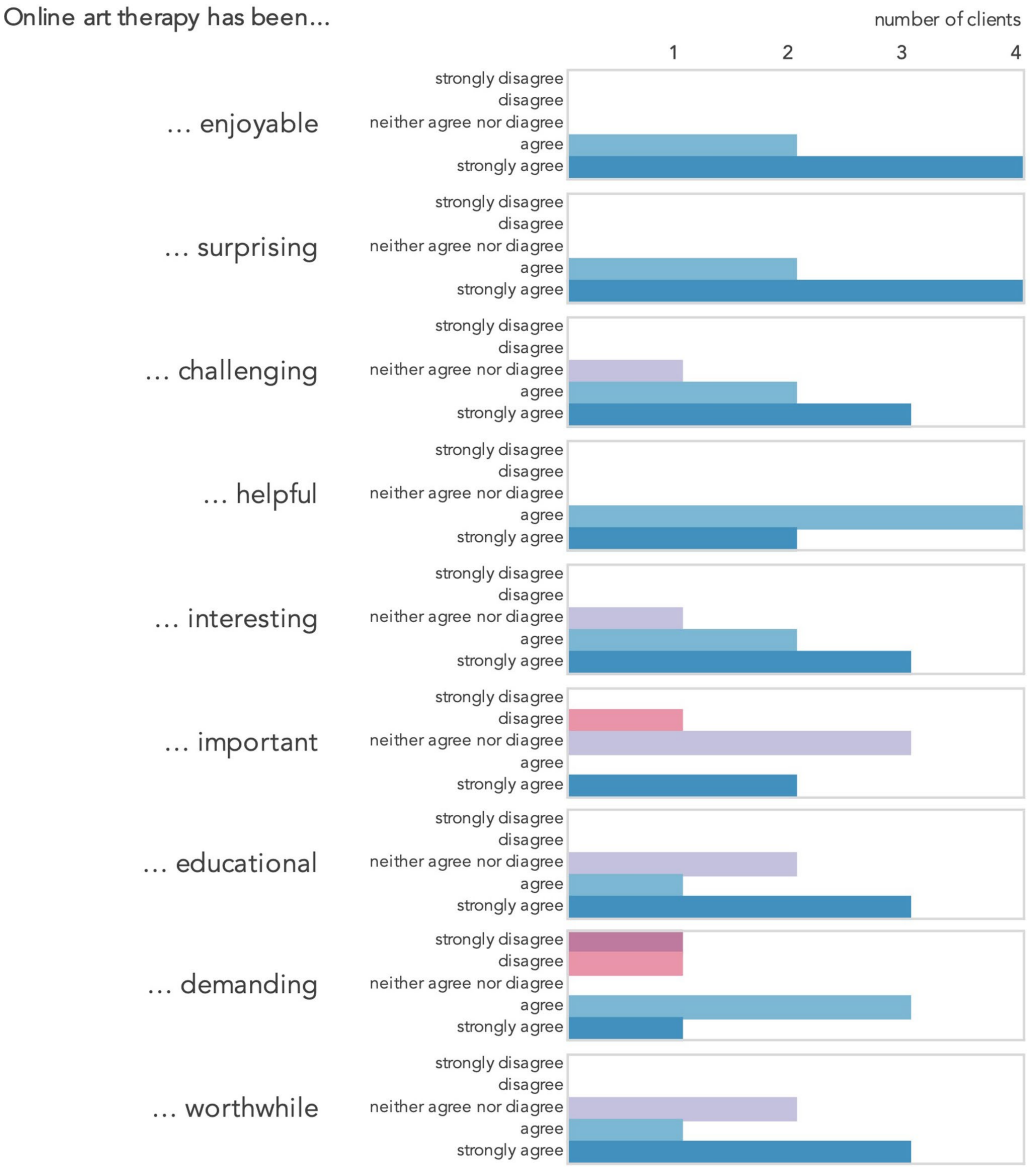
Responses to the post-therapy survey item 17: How strongly do you agree or disagree with the following descriptions of your online art therapy experience?

Clients who took part in the post-therapy survey agreed to varying degrees that the experience of art therapy had been enjoyable, surprising and helpful (Figure 2). The majority also indicated that it was interesting, educational and worthwhile, but also challenging. Respondents differed in their opinions on whether the therapy was important and demanding with more respondents leaning towards agreement rather than disagreement with these descriptors. Three respondents felt that the above words did not describe their experience fully and complemented them with their own, which included: *relaxing, thought provoking, enlightened, fulfilling, revealing of different perspectives* and *a safe and nurturing space*, which *made you make time for yourself (Cs)*.

#### 3.3.2. Nature of online art therapy

Art therapists felt that their clients had a good understanding of the nature of the art therapy process and recognised that it is a very different experience to verbal psychotherapy, counselling, art classes or a conversation with a friend [*It’s not like having a cup of tea with somebody, you can still have a conversation but it’s pulling things out*… *(C)*]. Most of the clients have had previous experience of psychological therapy or counselling and were able to compare those with their experience of art therapy, noticing the unique role of artmaking, non-verbal expression and the therapeutic relationship involving the therapist, the client and, to some extent, the artwork: *Everybody was able to make that difference in terms of how that process of nonverbal plus that supportive therapeutic relationship really allowed them to use the session (AT).*

Comparing their experience of online art therapy to seeing a doctor or a psychiatrist, participants highlighted insufficient time during doctor appointments to *talk about everything properly (C)* and that art therapy offered a more personal and dialogue-encouraging person-centred approach: *It’s good to talk to somebody and you feel you get feedback. I do not feel like I get feedback from my doctor because [they are] just dealing with certain issues that you go with (C).*

Clients who have had previous experience of other psychological therapies appreciated the opportunity for non-verbal expression and communication in art therapy, which to some felt less restrictive and relieving pressure of having to be precise in describing often difficult and complex emotions and problems: *[Other therapies] are often quite structured and they involve a lot of talking. So it’s nice just to not always talk, just see what the non-verbal part of you is saying. (C); You’re not having to be concerned about how it is you are explaining things whereas you do to a certain degree when you are just sitting across the table and you are chatting with that person. (C)* Some clients recognised the role of artmaking and the image in being able to progress in their therapy: *In talking with someone you need to be aware of how you word your feelings*… *With the artwork you are free to let your mind think what it wants (C)*.

Without the images, you'd just be sitting there trying to put all these emotions into words and sometimes there aren't good words to really convey everything simultaneously. You’re feeling so many things and you're trying to process it and you're trying to think about how to explain it to someone but with art therapy, you just put a brush on paper and you just see where it goes. You don't have to worry about articulating in any specific way. Taking that pressure away is just so beneficial. (C)

One person compared their experience of art therapy to art classes they used to attend throughout their life, recognising that they did not offer the therapeutic benefit they hoped for:

Once you do art therapy in that supportive, nurturing environment with a professional, you realise what you've been missing trying to just do art in an academic setting, it just doesn't have anywhere near the same effect. (C)

Feeling safe in the therapy space was essential for clients and something they particularly appreciated [*With art therapy it is just such an open-minded experience and such a nurturing experience, it’s very gentle, you cannot really do it wrong. (C); It’s just a really safe environment to explore things that you do not even realise are inside you (C)*]. One client recognised that feeling safe was a prerequisite to therapeutic progress, while it might not seem obvious: *I think it sometimes takes a little bit for people to adjust to that, to realise that they are safe in this little space. And I think it’s really interesting to see what happens as that develops (C).*

**Figure 3:**
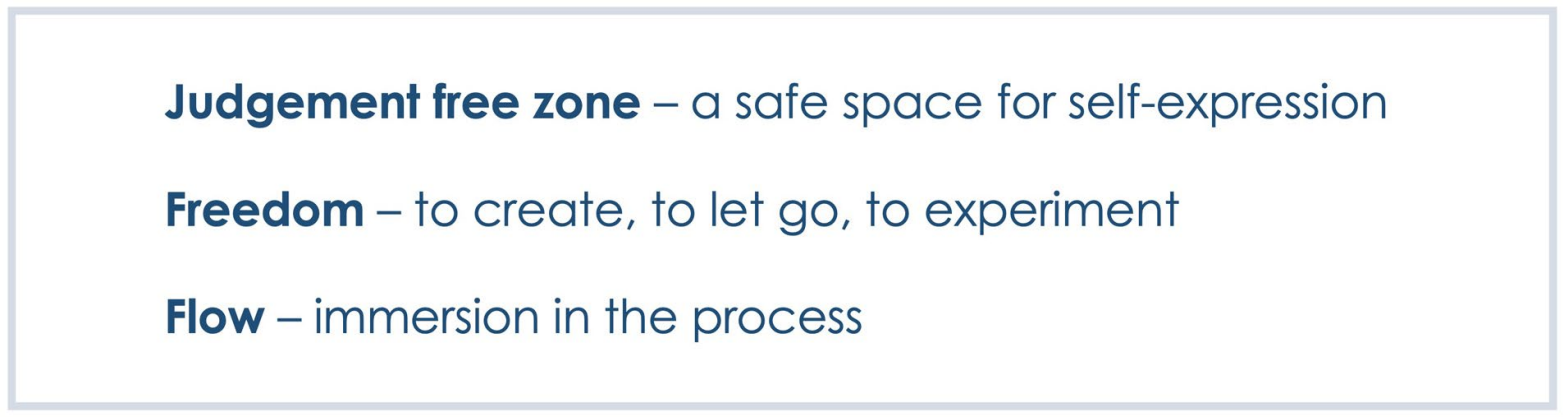
The three distinctive features of online art therapy as identified by clients.

Within the unquestionable frame of the therapeutic relationship, non-verbal expression and feeling safe, three distinctive though interconnected themes have clearly emerged from how participants described their experience in their own words (Figure 3). They seem to encapsulate the features of online art therapy which the clients felt were important and, supposedly, differentiating online art therapy from treatments they had received previously.

One client described an online art therapy space as a *judgement free zone* (C), which seemed to have echoed across participants’ experience and was also noticed by staff: *[The client] felt that [they] could express [themselves] and not be worried about being judged (S)*. One client described their relief on realising that not only would they not be judged in the therapy space, they themselves did not need to apply judgement to their artmaking: *It’s made me realise that I do not have to be so hard on myself when it comes to painting or drawing. It does not have to be a perfect reflection of whatever it is that you are trying to draw (C)*.

Most clients referred to the liberating feeling of freedom that art therapy allowed [e.g. *Making the art piece left you free to let your mind wander as to where it could take you (C). Just being able to let go and be free and just try these materials out and actually just go with it (C)*]. For one client the feeling of freedom was linked with expanding mind: *The art therapy is giving you more freedom* (…) *it’s making you expand more, it’s making you think more (C)*. This freedom to experiment seemed to have extended to portraying and accepting a range of emotions:

You’re having to think about other things around you that you can maybe pull into the artwork, things that are going on in life, you can put them down (…) you can either be wild with it or you can be sad with it or you can be happy with it (C).

One staff participant felt that the freedom their clients felt was related not only to creative expression but equally to being able to communicate freely with the therapist: *It was the freedom around the art side but around the conversation as well, total freedom, opportunity to vent, to share (S).*

Some clients also seemed to have referred to enjoying a state of creative flow while engaging in art making, describing a lost sense of time and feeling fully immersed in the process: *A few times when I’ve been working on an artwork I’ve lost track of time because I am getting stuck with what I’m doing and I’m enjoying myself (C); I get lost in my pictures and I feel like I’m actually there (C).*

#### 3.3.3. Benefits from online art therapy

A range of benefits for psychological wellbeing were observed by all involved in the pilot, including the clients themselves. One staff participant emphasised that they *found this project to be the most beneficial for [their] clients (S)* in relation to other psychological therapies and social prescribing activities they took part in previously. One participant thought that *it was a surprise to [clients], how much they got from it (S).*

One participant felt that art therapy was particularly helpful for anxiety *because it forces you to focus on something else, you have to concentrate on what you are creating in the present moment (S).* One client confirmed that *art therapy was great for helping [them] keep focused (C).* A staff participant also recognised that *the therapist was so patient with [them], let [them] work at [their] own pace, let [them] speak when [they] felt like speaking, which had a very calming effect on [them] (S).* Another staff participant felt that the therapy offered benefits that lasted beyond its duration: *It was overwhelmingly positive reactions from people, what they got from it, at the time of the sessions and ongoing (S).*

Of the six clients who completed the post-therapy survey, four reported that they had noticed benefits for themselves and two were not sure. One person felt that they had become more acceptant and observing of own feelings. Another client felt that they had learnt about art therapy and were *able to support [themselves] better* (C). One client felt *strong and resilient (C)* and another felt *more happy (C).* One of the clients who were not sure if they had noticed benefits, explained that they found themselves in an overly busy time during their therapy due to unforeseen circumstances, but the therapy made them realise that they *needed time for just [themselves] (C).*

**Figure 4:**
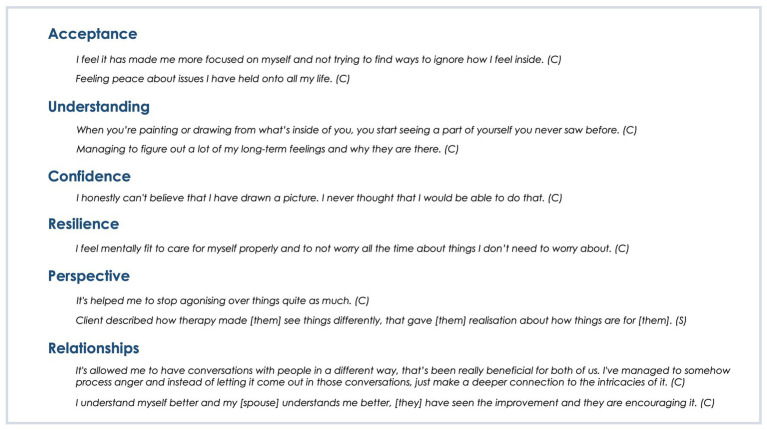
Areas of indicated benefits for wellbeing.

The clients indicated several areas of psychological wellbeing which they felt were impacted by their art therapy (Figure 4), including (a) acceptance - of emotions and things that cannot be controlled, (b) better understanding of self, (c) increased confidence, (d) improved resilience, (e) a newly gained perspective on aspects of life and (f) improved relationships.

A small sample in this study does not allow for statistical analysis of change on neither of the psychometric scales used. However, both scales have shown to be responsive to change at an individual level: for WEMWBS, a minimally important level of change in wellbeing was detected at 3 points difference (44) and for WHO-5 a 10% difference indicates a significant change (45). According to these estimates, between the two points in time, pre- and post- therapy, positive changes in wellbeing could be observed on both scales for one of the three clients for whom establishing and comparing scores was possible—with 12 points improvement in WEMWBS and 52% improvement in WHO-5 (Participant 1, Table 1). The results were mixed for the other two clients, with both negative (on WEMWBS) and positive changes (on WHO-5) detected for one client (Participant 3, Table 1), and negative change only indicated for another client on WHO-5 (Participant 2, Table 1).

**Table 1:** WEMWBS and WHO-5 scores for the three participants who completed both questionnaires.

	WEMWBS		WHO-5	
	Pre-therapy	Post-therapy	Pre-therapy	Post-therapy
Participant 1	44	56	40	92
Participant 2	50	48	80	40
Participant 3	47	43	40	52

#### 3.3.4. Beyond online art therapy

Some impacts of art therapy clearly lasted beyond the time in which it was offered. One art therapist felt that their client’s *realisation that she needs this for herself (AT)* was an important outcome for that client and might possibly allow them to take actions towards improved wellbeing. One staff participant shared that *when [their client is] feeling down, [they] look regularly at what [they have] already created. Through therapy [they] have found a way to do something positive (S).* One client concluded: *I still feel a little bit trapped, but from how I feel now to when I started the first session, it’s just a big improvement (C).*

Of the six clients who took part in the post-therapy survey, five said that they would recommend online art therapy to others and one person was not sure. Clients felt that more people would be interested in art therapy if it was available to them and they were already recommending it to people they know: *There’s so many people needing it, people I’ve spoken to*… *I want people to know about it and how good it is (C).*

Inspired by art therapy experience, some clients developed interest in group activities and art classes which they may be able to attend having regained *confidence to try out other things (S)*. One client went to an art event which was not something they would have done before and which the referrer felt *was like this has opened that door for them (S)*. Some clients found new (or rediscovered) interest in developing own artistic practice with one participant expecting that they would *probably continue with the art [themselves] (C),* another noticing *the urge to continue making art pieces (C)* and one person even considering creating a dedicated artmaking space in their home. One staff participant noticed that two of their clients *were still using art materials they got left (S)* and an art therapists observed that artmaking became an important part of their client’s daily life, *it totally took off in [their] life (AT)*:

There was a real sense of people continuing with the art after [art therapy] and talking about it in terms of art making as opposed to art therapy for themselves afterwards, really wanting to keep that connection with their art making process, that feeling. (AT)

Staff participants felt that not many other services in the Western Isles would be able to address the clients’ needs identified through this pilot. Some psychological therapies were restricted to specific age groups or specific geographical locations only. Art therapy was available face-to-face but with limited reach and demand exceeding capacity. Clients themselves found the online art therapy service valuable and felt that others would also find it beneficial:

Just to encourage it to continue or to be on offer somehow, so that people can access it (…) because without it so many people are just left out and not managing to live a quality of life really (C). This is a service that is so important. It has turned my life around (C).

## 4. Discussion

This pilot feasibility study highlighted priorities and considerations for future (online) art therapy practice and its evaluation. It confirmed the notion from previous research that safe and effective online practice demands adaptations in relation to traditional face-to-face therapy (25, 33) and demonstrated that online art therapy can be an acceptable intervention, much valued by clients. It also provided one of the first documented illustrations of how online art therapy practice presents unique opportunities for the therapy process and should not be seen as simply replicating traditionally used methods in an online setting (26).

Setting up a successful online art therapy service, therefore, requires not only practical and technological considerations but, most importantly, demands a good understanding of what it can offer from multidisciplinary teams involved in referrals and ongoing client support. While art therapy practice retains much of its fundamental nature in an online setup, it is important to recognise that achievable therapeutic aims might, in some cases, differ from those expected in face-to-face therapy. This might mean reconsideration of client suitability for therapy, including increased relevance for some clients who might not otherwise use face-to-face services, but also careful reassessment of clients for whom online delivery is less likely to work well. As online services are becoming increasingly common, confidence of referrers is likely to grow with increased familiarity and knowledge. In the meantime, ongoing dialogue between art therapists, service designers and providers is essential for a shared understanding of what is achievable and safe in an online setting.

This study demonstrated, as indicated previously for other online psychotherapies (38, 39), that the therapeutic process and relationship can both develop successfully in an online art therapy, sometimes in fact faster and more successfully. In some cases, a skilful art therapist can incorporate challenges of online connectivity into the therapeutic process, revealing aspects of personal difficulties which would be difficult to identify in a face-to-face setting. Previous studies similarly indicated links between the therapeutic progress and unique circumstances of connecting online, including in the intimacy of inevitably sharing images of a home space and glimpses of personal life (24, 25).

Therapeutic relationship is clearly possible in an online art therapy setup, as confirmed by both the clients and the therapists in this study, and it is evident that it takes the triangular form unique for the art therapy practice (or perhaps hexagonal, as proposed more recently (23), with the artwork and artmaking process just as important as the relationship between the two humans involved, and perhaps in fact intensified in an online setting (24). However, it needs to be recognised that developing a successful therapeutic relationship online demands, at times, a particularly skilful facilitation from an art therapist (33, 46), including an increased awareness, focus and effort. Creating a safe space for sharing and reflecting, which is core to art therapy practice, is made more challenging in an online setup, where the art therapist needs to support the client remotely in being creative and protecting this space for themselves. This process is not currently adequately supported by online communication platforms and art therapists often need to demonstrate creativity and resourcefulness in identifying solutions that work best for individual clients (47).

While this feasibility study aimed primarily to increase understanding of acceptability, suitability and mechanisms of online art therapy, it also provided rare insight into wellbeing-related benefits for individual clients, as perceived by clients themselves and observed by staff involved in their care. Online art therapy as offered in this study was reported to result in increased acceptance of emotions, understanding of self, a new perspective, confidence and resilience, all of which are outcomes observed routinely in art therapy practice [e.g. (8, 12, 48, 49)]. Parallels with what would be expected in a face-to-face therapy suggest the potential of online art therapy to achieve comparable outcomes, despite some differences in how the therapeutic process develops. Mechanisms that contributed to these positive impacts were identified by clients as: (a) non-judgemental approach, (b) freedom of expression, and (c) immersion in the process, which the clients felt were not necessarily achievable within other health services or, indeed, other forms of psychotherapy. In fact, the emphasis that clients put on reporting of feeling safe and free to express themselves and explore emotions and thoughts, both verbally and creatively, as opposed to in other therapy situations, suggests potential value of online mode of delivery itself in offering greater perceived intimacy and freedom of expression, at least for some clients.

Aside from a small number of participants in this study, a key limitation from the research perspective was its non-directiveness in terms of the art therapy approach. Due to variability within the intervention, it was not possible to develop an understanding of active ingredients (50) in the online art therapy process. However, the same quality ensured ‘real life’ testing of the online art therapy service, retaining as many elements of authentic practice as was possible, which, supposedly, allowed for insights truly aligned with local needs. Similarly, incorporating a range of methods might have arguably decreased precision of data collection and reporting. It has, however, allowed for a person-centred experience for clients and staff as research participants, resulting in depth of insights and reflection, including on acceptable and meaningful research procedures, which is expected to strengthen the research design of future studies.

Online art therapy, as demonstrated in this study, can be a relevant and welcomed intervention for adults living in rural and less populated areas. While careful consideration needs to be given to the service setup, coordination of a multidisciplinary team and ensuring safety and comfort for clients, potential benefits seem to outweigh effort. Future research could further explore the indication of the therapeutic value of online spaces within art therapy and beyond. There remains more to be discovered around the potential that connecting online offers to the therapeutic process for all psychotherapies and for art therapy in particular, given the unique role of an image within the therapeutic relationship. As this study raised a question about usefulness of common mental wellbeing measures within the island community context, more research is recommended into most culturally appropriate tools for measuring change and inter-intervention comparison (51, 52). Further studies should aim to increase our understanding of the relevance of online art therapy for rural communities and propose ways in which online interventions would best meet the local mental health needs and how they would best fit within the wider mental health provision.

## Data availability statement

The datasets presented in this article are not readily available because data contains sensitive personal material of psychotherapy clients and is not suitable for public sharing due to potential for identification of participants from a small island community. Requests to access the datasets should be directed to AZ, ania.zubala@uhi.ac.uk.

## Ethics statement

This study involving human participants was reviewed and approved by the Research Ethics Committee of the University of the Highlands and Islands and the NHS Western Isles R&D. The clients and staff participants provided their written informed consent to participate in this study.

## Author contributions

AZ, NK, CM, MMac, and MMal contributed to conception and design of the study. AZ, NK, and CM undertook the research. MMac supported data collection. AZ wrote the first draft of the manuscript. All authors contributed to the article and approved the submitted version.

## Funding

This pilot service and its evaluation were funded by the NHS Western Isles EU INTERREG VA mPower project.

## Conflict of interest

The authors declare that the research was conducted in the absence of any commercial or financial relationships that could be construed as a potential conflict of interest.

## Publisher’s note

All claims expressed in this article are solely those of the authors and do not necessarily represent those of their affiliated organizations, or those of the publisher, the editors and the reviewers. Any product that may be evaluated in this article, or claim that may be made by its manufacturer, is not guaranteed or endorsed by the publisher.
